# Subjective Efficiency Evaluation after Maxillomandibular Advancement Surgery in Obstructive Sleep Apnea Patients

**DOI:** 10.3390/jcm12124023

**Published:** 2023-06-13

**Authors:** Karel Kuik, Misha L. Tan, Jean-Pierre T. F. Ho, Jerôme A. H. Lindeboom, Jan de Lange

**Affiliations:** 1 Department of Oral Maxillofacial Surgery, Amsterdam University Medical Center, Location AMC, 1105 AZ Amsterdam, The Netherlands; 2 Department of Oral Maxillofacial Surgery, Noordwest Ziekenhuisgroep, 1815 JD Alkmaar, The Netherlands; 3 Department of Oral Maxillofacial Surgery, Ziekenhuis Amstelland, 1186 AM Amstelveen, The Netherlands

**Keywords:** maxillomandibular advancement, obstructive sleep apnea, quality of life

## Abstract

Purpose: To investigate subjective efficiency outcomes after maxillomandibular advancement (MMA) surgery in obstructive sleep apnea (OSA) patients. Material and Methods: A prospective cohort study was carried out between December 2016 and May 2021, including 30 severe or treatment-refractory OSA patients treated by MMA surgery. All patients answered four validated questionnaires: the Epworth Sleepiness Scale (ESS), Functional Outcomes of Sleep Questionnaire (FOSQ), Mandibular Function Impairment Questionnaire (MFIQ), and EQ-5D-3L (i.e., EQ-5D and EQ-VAS). They also answered one custom-made questionnaire (AMCSQ). Questionnaires were requested to be filled out 1 week before surgery and at least 6 months after surgery. Results: The total preoperative and postoperative scores on the questionnaires were compared. The mean total ESS (*p* < 0.01), FOSQ (*p* < 0.01), EQ-5D (*p* < 0.05), and EQ-VAS (*p* < 0.01) scores showed significant improvement, which was in accordance with an improvement in the mean postoperative apnea/hypopnea index score (*p* < 0.01). In contrast, the mean total MFIQ score (*p* < 0.01) indicated a decline in mandibular function. Conclusion: This study confirms the hypothesis that MMA surgery in OSA patients improves outcomes, both objectively and subjectively, with the exception of postoperative mandibular function.

## 1. Introduction

Obstructive sleep apnea (OSA) is a common disorder that can impair daily life and affect health, cognitive function, and state of mind [1,2,3]. OSA has a large global disease burden, with a prevalence of 9–38% estimated in the general population that increases with age [4]. Multiple possible treatments have been described, from noninvasive treatments to multilevel surgery [5].

Maxillomandibular advancement (MMA) surgery is the most successful surgical treatment, aside from tracheostomy [6,7,8,9]. MMA includes a large forward advancement of the maxilla and mandible to increase the upper airway volume and put more tension on the parapharyngeal soft tissue [7].

Many studies have focused on objective surgical outcomes measured during polysomnography (PSG): the apnea/hypopnea index (AHI), position dependency, and central and mixed apnea [7,10,11,12]. There is, however, increasing discussion on the role of the AHI, which is used as the standard measurement for defining the severity of OSA [13]. Modifications to the American Academy of Sleep Medicine (AASM) scoring system for hypopneas have resulted in changes in the AHI score and prevalence of OSA [14]. However, the AHI may not fully define the clinical features of OSA patients [13]. Given the greater focus on the patient and less on the numbers, the characterization of OSA patients should comprise not only PSG measurements, but also subjective outcomes regarding daytime sleepiness, functional status, and quality of life (QoL).

This study assessed the subjective outcomes related to daytime sleepiness, mandibular function, sleep-related QoL, and general health in OSA patients undergoing MMA surgery using multiple validated questionnaires [15,16,17,18]. 

## 2. Materials and Methods

### 2.1. Patients

This prospective cohort study was conducted between December 2016 and May 2021. Patients with severe OSA or treatment-refractory OSA referred to the Amsterdam University Medical Centers (location Academic Medical Center) for MMA surgery were eligible for participation in this study. Adult patients (age ≥ 18) with sufficient command of the Dutch language were included. Patients with previous history of orthognathic surgery were excluded. Twenty of the 30 patients included in this study previously had other upper-airway treatments (uvulopalatopharyngoplasty, tonsillectomy, nasal septoplasty, or Celon treatment) for OSA elsewhere, but without any satisfactory results. Patients eligible for MMA provided informed consent and were asked to complete four validated questionnaires and one custom-made questionnaire 1 week prior to surgery and at least 6 months after surgery. The study was approved by the Medical Ethical Committee of the Amsterdam University Medical Center (reference W17_237). 

### 2.2. Evaluation of Medical Records

Patient characteristics (age, gender, and BMI) and comorbidities (diabetes mellitus and cardiovascular diseases) were obtained from standardized intake medical records. Preoperative and postoperative PSG data, AHI, central apnea index (CAI), mixed apnea index (MAI), lowest oxygen saturation (LSAT), oxygen desaturation index (ODI), and cephalometric data (SNA, SNB, ANB, and degree of advancement of the A-point, B-point, and pogonion) were used to measure objective outcomes. 

PSG (level 1 or level 2 examination) was performed at several centers preoperatively and at least 3 months postoperatively. PSG data were extracted according to the standards of the AASM Manual for the Scoring of Sleep and Associated Events [19]. 

Perioperative and postoperative complications were identified by evaluating the medical records. Surgical success was defined as postoperative AHI changes ≥ 50% and < 20 events/hour, as defined by Sher et al. [20].

### 2.3. Questionnaires 

The patients were asked to complete four validated questionnaires on the online electronic data capture platform Castor EDC between December 2016 and May 2021 to assess the subjective outcome [21]. Before these questionnaires, a short, custom-made questionnaire (AMCSQ) about OSA complaints was completed, including whether the patient snores and how affected patients’ partners were by snoring on a scale of 0 to 10 (AMCSQ added as Supplementary Material). 

The Epworth Sleepiness Scale (ESS) is a validated psychometric questionnaire in which subjects are instructed to rate how likely they are to doze off or fall asleep in different situations on a scale of 0 to 3 [15]. The total score categorizes the severity of the patient’s daytime sleepiness [15].

The Functional Outcomes of Sleep Questionnaire (FOSQ) is utilized to assess the impact of disorders of excessive sleepiness on multiple activities of everyday living [16]. The questionnaire is divided into five subscales: activity level, vigilance, intimacy and sexual relationships, general productivity, and social outcome. On a 4-point rating scale, patients could rate to what degree sleepiness affects their daily functioning. The mean of all subscale scores can be multiplied by the number of subscales to obtain a total score. 

Mandibular function was assessed by the Mandibular Function Impairment Questionnaire (MFIQ) [17]. The MFIQ reliably assesses the degree of impairment of specific jaw functions without measuring the symptoms and signs causing the functional impairment. For each question, on a scale of 0–4, patients rate how much difficulty they have with a specific activity due to jaw complaints [17]. The sum score ranges from 0 to 68, with higher scores indicating greater restriction of the jaw.

The EQ-5D-3L questionnaire was used to assess the patient’s health status [18]. In the first part of the questionnaire, patients were given five questions to define their health on a 3-point scale representing five dimensions: mobility, self-care, usual activities, pain/discomfort, and anxiety/depression [18]. A summary index score, the EQ-5D index, can be calculated with weights based on the Dutch population. The index score ranges from less than 0 (worse health state) to 1 (perfect health). In the second part of the questionnaire, patients were asked to define their general health on a visual analogue scale (VAS), ranging from the worst possible to the best possible health state.

### 2.4. Statistical Analysis

The Shapiro–Wilk test was applied to verify the data distribution and normality. Nonparametric tests were used because the data were not normally distributed: 1. The Wilcoxon signed-rank test was used to compare the preoperative and postoperative scores of the ESS, FOSQ, MFIQ, EQ-5D index, and EQ-VAS. 2. The Spearman rank correlation coefficient was used to analyze correlations between PSG-, cephalometric data, and the questionnaires. 3. The McNemar test was used to assess differences in snoring before and after surgery. A *p*-value of 0.05 was considered significant. SPSS software (version 26.0, IBM Corp., Armonk, NY, USA) was used for statistical analyses. 

## 3. Results

### 3.1. Patient Characteristics

A total of 36 patients were asked to be enrolled in the study. Four patients did not respond to the questionnaires, and postoperative PSG data were missing for two patients. Moreover, two of these six patients were treated with monomaxillary surgery. Therefore, these six patients were excluded, and the final data set was based on 30 patients with a mean follow-up of 12 months. One patient did not complete the MFIQ postoperatively, and one patient did not complete the FOSQ, MFIQ, and EQ-5D-3L postoperatively. The mean patient age was 51.6 ± 10.4 years (range 31–70 years). Most patients were men (66.7%) and overweight (preoperative BMI, 28.3 ± 4.4 kg/m^2^). Five (16.7%) patients had cardiovascular disease, and three patients had diabetes mellitus (10.0%) (Table 1). The mean baseline PSG findings showed a preoperative AHI of 49.4 ± 25.1 events/hour, CAI of 1.8 ± 3.4, MAI of 8.9 ± 16.3, ODI of 54.1 ± 26.5 events/hour, and LSAT of 75.6% (Table 2). 

### 3.2. Surgical Outcomes

The mean degree of advancement of the A point (maxilla), B point (mandible), and pogonion (chin) was 6.3 ± 1.8 mm, 10.6 ± 3.6 mm, and 10.7 ± 4.5 mm, respectively. The postoperative PSG showed significant improvement in the mean AHI (*p* < 0.01), MAI (*p* = 0.04) ODI (*p* < 0.01), and LSAT (*p* < 0.01) after MMA (Table 2). MMA tended to improve CAI, but the difference was not significant (*p* = 0.09). The surgical success rate in the study population was 56.7%.

### 3.3. Subjective Outcomes

The ESS, FOSQ, MFIQ, and EQ-5D-3L scores are provided in Table 3. The mean ESS score decreased significantly from 12.3 to 5.8 (*p* < 0.01), indicating a significant improvement in subjective sleepiness. Improvements in the ESS were significantly associated with the AHI, ODI, and LSAT improvements (R = 0.51, *p* = 0.01; R = 0.66, *p* < 0.01; and R = 0.59, *p* < 0.01, respectively). 

The mean FOSQ score increased significantly from 14.2 to 16.8 (*p* < 0.01), indicating improvement in the sleep-specific QoL. The increase in FOSQ score was significantly associated with the AHI and ODI (R = 0.43, *p* = 0.02, and R = 0.55, *p* < 0.01, respectively). Furthermore, the FOSQ significantly improved in four out of five functional domains (general productivity, social outcome, activity level, and vigilance). No significant difference was found in the domain of intimate relationships and sexual activity (*p* > 0.05). 

The mean MIFQ score increased significantly from 6.9 to 15.0 (*p* < 0.01), indicating worse mandibular function after MMA. The change in the MFIQ score was significantly associated with the degree of advancement of the A point (R = 0.39, *p* = 0.04). However, it was not related to the degree of advancement of the B point and pogonion or the PSG parameters (*p* > 0.05).

The EQ-5D-3L showed improvement in both the EQ-5D index and the EQ-VAS score. The baseline EQ-5D index was 0.70 ± 0.24 and it increased to 0.78 ± 0.22 (*p* < 0.05). The EQ-VAS score increased from 54.7 ± 18.5 to 68.3 ± 18.6 (*p* < 0.01). The change in the EQ-VAS score was significantly correlated with AHI improvement (R = 0.38, *p* < 0.05).

Subjective self-assessment of snoring significantly decreased in patients who snore (*p* < 0.01). Thus, the mean score of how affected the partner is by snoring also decreased (*p* < 0.01) (Table 4).

## 4. Discussion

This study aimed to assess the subjective outcomes of patients undergoing MMA surgery to treat OSA. Four questionnaires were used to investigate various subjective aspects, namely the ESS (sleepiness), MFIQ (mandibular function), FOSQ (sleep-specific QoL), and EQ-5D-3L (general health state). Thirty patients underwent MMA surgery and were followed up for at least 6 months, with a mean follow-up of 12 months. The mean postoperative AHI, MAI, LSAT, and ODI significantly improved compared to baseline. The surgical success rate was 56.7% [20], which is considerably lower than in earlier reports (pooled rate of 85%) [8]. A possible explanation could be selection bias. Many patients in this study were refractory to multiple therapies before engaging in maxillomandibular surgery. In addition, some of the patients suffered from central apnea or cardiovascular disease, which are associated with non-response to MMA for OSA [22]. 

The current study found a significant improvement in subjective sleepiness with a mean postoperative ESS score of 5.8 ± 4.2, which indicates normal levels of sleepiness after the operation (ESS score ≤ 10). This result is consistent with other MMA studies reported in the systematic review and meta-analyses by Zhou et al. [8]. 

The sleep-specific QoL improved significantly after the operation, but the postoperative FOSQ score was 16.8 ± 3.2, which indicates a not completely normal functional status (FOSQ score ≥ 18) [16]. 

The mean improvement in the total FOSQ score was more significant in other studies with a similar follow-up [23,24]. An explanation for this difference may be the surgical success rate, which was higher than in the current study [23,24]. However, in contrast to this study, Boyd et al. excluded patients with central and complex sleep apnea [24]. CAI was not significantly improved after MMA in the patients in the current study. This may explain the lower FOSQ and ESS scores. 

The preoperative MFIQ score of 6.9 indicates that patients hardly ever experience problems with function before surgery. A slight increase was found in the MFIQ score after the surgery, to 15.0, implying worse mandibular function than the baseline MFIQ score. This could be explained by it taking time to get used to the advancement of the jaw after MMA surgery. In addition, the postoperative questionnaires were completed six months after surgery in some patients. Therefore, it is possible to think that the postoperative complications, including malocclusion, facial numbness, and jaw stiffness, had not disappeared completely at that time [7]. Furthermore, mandibular nerve injury is a common postoperative complication and has been reported to resolve in 85–90% of patients by six to twelve months [25]. 

A decrease in oral health-related QoL (OHRQoL) was seen in the immediate postoperative period, though, this improved after six months and one year [26]. Moreover, some patients were treated with orthodontic appliances for a considerable preoperative and postoperative period [27]. This may influence the (OHRQoL) [28]. According to a meta-analysis by Yi et al., the OHRQoL of orthognathic patients improves during postsurgical orthodontic treatment (six months after surgery), surpassing the scores of the presurgical orthodontic treatment [28]. Patients reported improved oral function (OQoL-22 questionnaire subscale) six months after conventional combined orthodontic–surgical treatment compared to before surgery [28]. In this study, the authors preferred to evaluate subjective mandibular function using the MFIQ. Although both questionnaires are associated with oral function, the MFIQ is solely focused on oral function with a more detailed approach. No other MMA studies have used the MFIQ to measure mandibular function. Although earlier studies showed a correlation between the degree of maxillary advancement and the decrease in AHI, the results of this study emphasize the balance between the amount of maxillary advancement and the decrease in mandibular function, as seen in the correlation between the MFIQ score and the degree of advancement of point A [7]. Surgeons should inform patients about this possible downside of the operation. 

General health, measured by the EQ-5D-3L, was expressed by the EQ-5D index and EQ-VAS score. Both scores improved significantly after MMA compared to baseline. The postoperative EQ-VAS score was lower than in the healthy general population in the Netherlands (EQ-VAS of 81.4) [29]. One study reported the EQ-5D-3L after MMA and found higher EQ-VAS scores in patients treated successfully with MMA than in the failure group [30].

This study possesses certain limitations. Firstly, this study was conducted in a specific patient population within a tertiary care academic hospital, limiting the generalizability of the results. Secondly, the sample size was small. In addition, two patients did not complete all the questionnaires during follow-up and were excluded from the analysis of these specific surveys. However, the authors chose not to exclude these patients from the study, due to the separated topics of the questionnaires and the small sample size. A possible explanation for the missing response is that patients were not motivated to complete all the questionnaires because of their length or number. Nevertheless, strong evidence for an association between the length of a questionnaire and response rate is lacking in the current literature [31]. Another limitation is related to the study design. While it was a prospective cohort study regarding questionnaires, the data on PSG and cephalometric measurements were collected retrospectively; therefore, some data were missing. Additionally, this study had a relatively short-term follow-up period, which may not accurately obtain long-term postoperative complications or outcomes. Lastly, a limitation of this study is that some of the sleep apnea patients in this study were refractory to previous therapies, which could influence the result of MMA surgery. Assessing subjective outcomes through repetitive questionnaires after each treatment and implementing questionnaires at the baseline diagnosis of sleep apnea could provide valuable insights. The authors suggest future long-term studies with larger sample sizes, more frequent questionnaires, and larger patient numbers to investigate the subjective outcome after MMA. Thereby, the authors advise multicenter research and a multidisciplinary approach, in which questionnaires are integrated into a clinical protocol for the care of OSA patients. Inclusion should ideally start with the first consultation after the initial referral. 

With the results of this study, surgeons will have better insight into patients’ perceptions of MMA surgery. Before surgery, a better expectation pattern can be created for the patients. After surgery, patients can be better supervised. In contrast to PSG variables, patients can be provided with more perceptions of daily function after surgery with subjective variables such as daytime tendency to sleep, sleep-related quality of life, general health, and mandibular function. 

## 5. Conclusions

The findings of this study show that MMA is a successful treatment for OSA patients when looking at subjective improvement in sleepiness, sleep-specific QoL, and general health state. However, postoperatively, patients experience a slight deterioration in mandibular function. 

## Figures and Tables

**Table 1:** Patient characteristics (*n* = 30).

Variable		Range
Gender (M/F)	20/10	
Age (years)	51.6 ± 10.4	31–70
Objective BMI pre op (kg/m^2^)	28.3 ± 4.4	18.0–34.6
Subjective BMI pre op (kg/m^2^)	28.5 ± 4.5	19.2–34.4
Subjective BMI post op (kg/m^2^)	28.3 ± 4.3	19.4–34.3
CVD	5 (16.7%)	
DM	3 (10.0%)	

Values are mean ± SD or *n* (%) unless otherwise noted. M: male; F: female; SD: standard deviation; BMI: body mass index; pre op: preoperative; post op: postoperative; CVD: cardiovascular disease. DM: diabetes mellitus.

**Table 2:** Polysomnography variables.

	Preoperative				Postoperative				
Variable	Mean	SD	Min	Max	Mean	SD	Min	Max	*p*-Value
AHI	49.4	25.1	6.4	93.5	17.4	11.7	2.7	47.0	<0.01
CAI	1.8	3.4	0.0	12.7	0.4	0.7	0.0	2.4	0.09
MAI	8.9	16.3	0.0	58.0	1.8	4.1	0.0	17.2	0.04
ODI	54.1	26.5	2.2	93.4	21.1	12.3	1.3	51.1	<0.01
LSAT	75.6	12.1	46	92	83.0	7.3	64	92	<0.01

SD: standard deviation; AHI: Apnea–Hypopnea index; CAI: central apnea index; MAI: mixed apnea index; DI: oxygen desaturation index; LSAT: lowest oxygen saturation.

**Table 3:** Questionnaire scores (*n* = 30).

	Preoperative				Postoperative				*p*-Value
	Mean	SD	Min	Max	Mean	SD	Min	Max	
ESS score	12.3	6.0	2.0	23.0	5.8	4.2	0	17	<0.01
FOSQ score ^a^									
General productivity	3.0	0.7	1.3	4.0	3.5	0.6	1.7	4.0	<0.01
Social outcome	2.8	1.1	0.8	4.0	3.5	0.8	1.6	4.0	<0.01
Activity level	2.6	0.9	1.0	4.0	2.9	0.7	1.1	3.6	<0.02
Vigilance	2.7	0.9	0.8	4.0	3.4	0.7	1.6	4.0	<0.01
Intimate relationships and sexual activity	3.2	0.8	1.6	4.0	3.4	0.9	0.8	4.0	0.06
Total score	14.2	4.0	6.2	19.8	16.8	3.2	8.6	19.6	<0.01
MFIQ score ^b^	6.9	10.4	0	33	15.0	12.5	0	40	<0.01
EQ-VAS ^a^	54.7	18.5	10.0	90.0	68.3	18.5	20	100	<0.01
EQ-5D index ^a^	0.702	0.241	0.1	1.0	0.777	0.222	0.1	1.00	<0.05

SD: standard deviation ^a^ *n* = 29, ^b^ *n* = 28.

**Table 4:** Reported snoring and severity of snoring according to partner (VAS) (*n* = 30).

	Preoperative	Postoperative	*p*-Value
No snoring	3	17	
Snoring	27	13	<0.01
Mean score ± SD	6.9 ± 3.3	2.5 ± 3.5	<0.01

Values are number of patients unless otherwise noted.

## Data Availability

The data that support the findings of this study are available from the corresponding author, [MT], upon reasonable request.

## Supplementary Materials

The following supporting information can be downloaded at: https://www.mdpi.com/article/10.3390/jcm12124023/s1. Questionnaire: AMCSQ.

## Abbreviations

AASM	American Academy of Sleep Medicine
AHI	Apnea/hypoapnea index
AMCSQ	Academic medical center sleep questionnaire
BMI	Body mass index
CAI	Central apnea index
CVD	Cardiovascular disease
DM	Diabetes Mellitus
ESS	Epworth Sleepiness scale
FOSQ	The functional outcomes of sleep questionnaire
LSAT	Lowest oxygen saturation
MAI	Mixed apnea index
MFIQ	Mandibular function impairment questionnaire
MMA	Maxillomandibular advancement
ODI	Oxygen desaturation index
OHRQoL	Oral health-related quality of life
OSA	Obstructive sleep apnea
PSG	Polysomnography
VAS	Viual analogue scale
QoL	Quality of life
