# Supplementary figures and images for: Subjective Efficiency Evaluation after Maxillomandibular Advancement Surgery in Obstructive Sleep Apnea Patients
Source: J Clin Med. 2023 Jun 13;12(12):4023. doi: 10.3390/jcm12124023 (PMC10299079; doi:10.3390/jcm12124023)

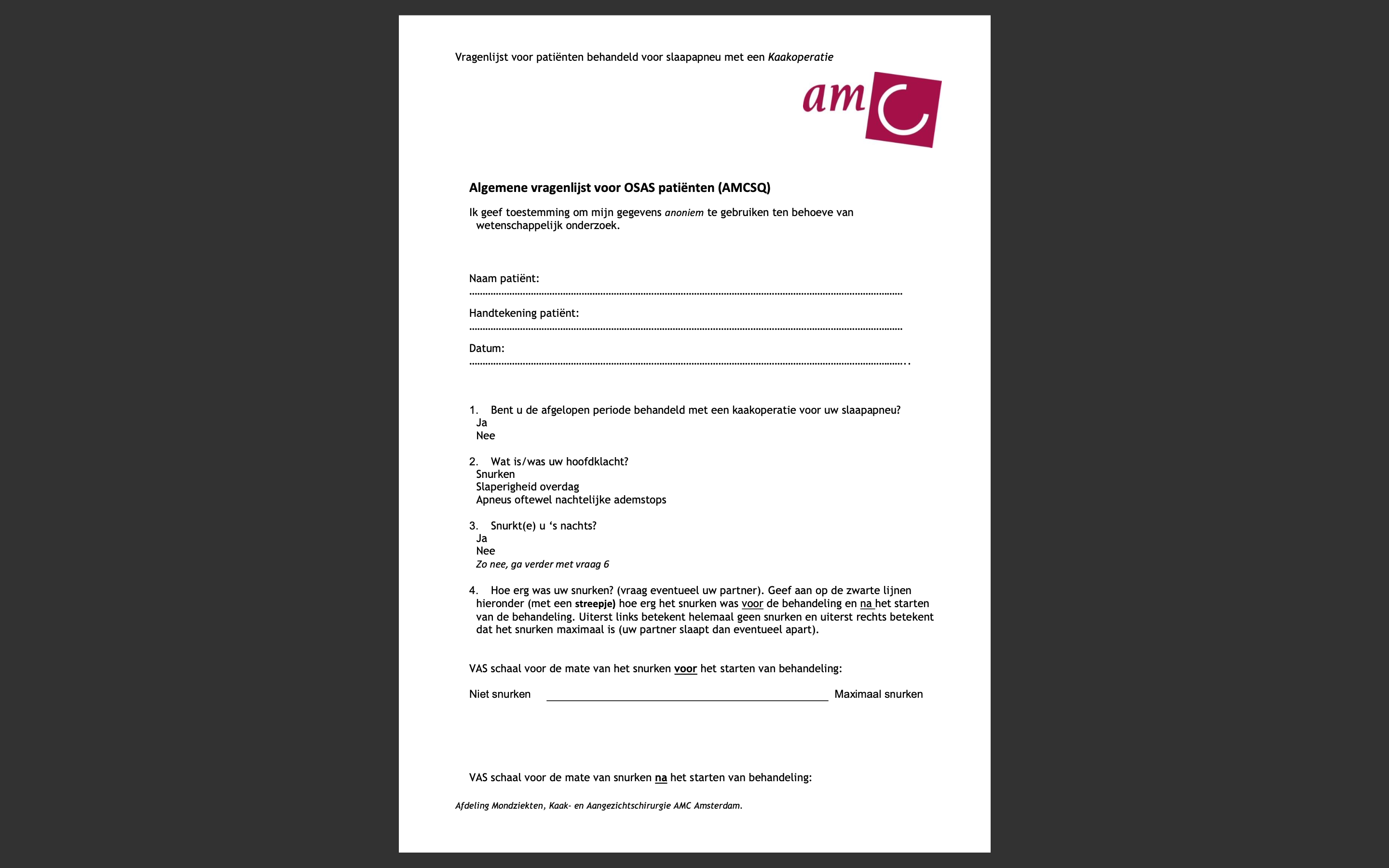

Supplement: Supplementary file 1 [file jcm-12-04023-s001.zip › jcm-2401357-supplementary.png]
